# Comprehensive analysis of tumor necrosis factor-α-inducible protein 8-like 2 (TIPE2): A potential novel pan-cancer immune checkpoint

**DOI:** 10.1016/j.csbj.2022.09.021

**Published:** 2022-09-17

**Authors:** Kun-Hao Bai, Yi-Yang Zhang, Xue-Ping Li, Xiao-Peng Tian, Meng-Meng Pan, Da-Wei Wang, Yu-Jun Dai

**Affiliations:** a State Key Laboratory of Oncology in South China, Collaborative Innovation Center for Cancer Medicine, 651 Dongfeng East Road, Guangzhou 500020, Guangdong, China; b Department of Endoscopy, Sun Yat-sen University Cancer Center, Guangzhou, 500026, China; c Department of Hematologic Oncology, Sun Yat-sen University Cancer Center, Guangzhou 500020, Guangdong, China; d Jiangsu Institute of Hematology, The First Affiliated Hospital of Soochow University, Suzhou 215006, Jiangsu, China; e National Research Center for Translational Medicine, Ruijin Hospital affiliated to Shanghai Jiao Tong University School of Medicine, 197, Ruijin Road II, Shanghai 200025, China

**Keywords:** LAML, Acute Myeloid Leukemia, BLCA, Bladder Urothelial Carcinoma, BRCA, Breast invasive carcinoma, CESC, Cervical squamous cell carcinoma and endocervical adenocarcinoma, CHOL, Cholangiocarcinoma, COAD, Colon adenocarcinoma, ESCA, Esophageal carcinoma, GBM, Glioblastoma multiforme, HNSC, Head and Neck squamous cell carcinoma, KICH, Kidney Chromophobe, KIRC, Kidney renal clear cell carcinoma, KIRP, Kidney renal papillary cell carcinoma, LIHC, Liver hepatocellular carcinoma, LGG, Lower Grade Glioma, LUAD, Lung adenocarcinoma, LUSC, Lung squamous cell carcinoma, *MESO*, Mesothelioma, OV, Ovarian serous cystadenocarcinoma, PAAD, Pancreatic adenocarcinoma, PCPG, Pheochromocytoma and Paraganglioma, PRAD, Prostate adenocarcinoma, READ, Rectum adenocarcinoma, SARC, Sarcoma, SKCM, Skin Cutaneous Melanoma, STAD, Stomach adenocarcinoma, TGCT, Testicular Germ Cell Tumor, THYM, Thymoma, THCA, Thyroid carcinoma, UCS, Uterine Carcinosarcoma, UCEC, Uterine Corpus Endometrial Carcinoma, UVM, Uveal Melanoma, TIPE2, New immune checkpoint, Pan-cancer, Stemness feature, Immune cell infiltration

## Abstract

Tumor necrosis factor-α-inducible protein 8-like 2 (TIPE2) is encoded by TNFAIP8L2 and is a newly identified negative regulator of natural and acquired immunity that plays a critical function in maintaining immune homeostasis. Recently, CAR-NK immune cell therapy has been a focus of major research efforts as a novel cancer therapeutic strategy. TIPE2 is a potential checkpoint molecule for immune cell maturation and antitumor immunity that could be used as a novel NK cell-based immunotherapeutic approach. In this study, we explored the expression of TNFAIP8L2 across various tumor types and found that TNFAIP8L2 was highly expressed in most tumor types and correlated with prognosis. Survival analysis showed that TNFAIP8L2 expression was predictive of improved survival in cervical-squamous-cell-carcinoma (CESC), sarcoma (SARC) and skin-cutaneous-melanoma (SKCM). Conversely, TNFAIP8L2 expression predicted poorer survival in acute myeloid leukemia (LAML), lower-grade-glioma (LGG), kidney-renal-clear-cell-carcinoma (KIRC) and uveal-melanoma (UVM). Analysis of stemness features and immune cell infiltration indicated that TNFAIP8L2 was significantly associated with cancer stem cell index and increased macrophage and dendritic cell infiltration. Our data suggest that TNFAIP8L2 may be a novel immune checkpoint biomarker across different tumor types, particularly in LAML, LGG, KIRC and UVM, and may have further utility as a potential target for immunotherapy.

## Graphical abstract

**Figure:**
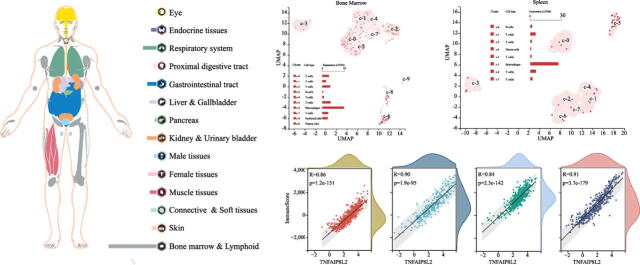

## 1 Introduction

Immunotherapy continues to revolutionize cancer treatments by activating the immune system to recognize and eliminate tumor cells [1]. Recent studies have focused on generating or releasing tumor antigen-specific T-cell responses and have provided a personalized treatment paradigm in several tumor types [2], [3]. Despite these advances, the clinical benefit of immunotherapy has been limited to subgroups of patients with specific tumor biologies [4]. Immunosuppressive signaling within the tumor microenvironment through inhibitory receptors on T-cells exert a suppressive anti-tumor T-cell response that contributes to the poor efficacy of conventional CAR-T in solid tumors [5], [6]. The molecular mechanisms of immune escape continue to be explored in greater detail with an increasing number of immune checkpoint inhibitors being investigated in clinical trials including anti-lymphocyte activation gene 3 (LAG3), anti-cytotoxic lymphocyte-associated protein 4 (CTLA4) and anti-programmed cell death protein 1 (PD-1) [1], [7], [8].

Natural killer (NK) cells are a critical component of the tumor immune response that have also been therapeutically exploited. CAR-NK cells are strong candidates for cancer retargeting therapy due to their unique mechanism of cellular recognition, high cytotoxicity and strong safety profile. CD56^bright^CD16^-^ and CD56^dim^CD16^+^ populations are the two most common subsets of NK cells. In the peripheral blood CD56^bright^ cells are less abundant where 90 % of the NK cells are CD56^dim^. However, in tissues, CD56^bright^ NK cells are the predominant cell population. Unlike B- and T-cells, NK cells do not express somatically rearranged antigen receptors but rather express a random combination of activating and inhibitory receptors. Similar to CAR-T cells, CAR-NK cells have extracellular, transmembrane and intracellular signaling domains. NK cells increase their cytotoxic capacity and levels of cytokine production through two other co-stimulatory molecules, NKG2D and CD244. Consequently, NK cells have a stronger capacity for tumor-specific targeting and cytotoxicity compared to CAR-T cells and so CAR-NK cell therapy is an attractive alternative to CAR-T therapy.

Tumor necrosis factor-α-inducible protein 8-like 2 (TIPE2) is encoded by TNFAIP8L2 and is a newly identified negative regulator of natural and acquired immunity that plays a critical function in maintaining immune homeostasis [9]. Mice with systemic TIPE2 deficiency are resistant to tumor growth suggesting that TIPE2 may suppress anti-tumor immune responses [10]. TIPE2 also promotes macrophage differentiation towards immunosuppressive M2 macrophages and is required for the immunosuppressive function of CD4^+^CD25^+^ Treg cells [11], [12]. TIPE2 has strong implications for the differentiation and function of immunosuppressive leukocytes. Studies have shown that TIPE2 is a potential checkpoint molecule for NK cell maturation and antitumor immunity [13]. At the population and individual cell levels, deletion of TIPE2 results in increased levels of mature NK subpopulations that have stronger effector functions [14]. Also, deletion of TIPE2 in NK cells significantly inhibits tumor cell growth *in vivo* and is accompanied by increased levels of tumor-infiltrating NK cells and increased expression of functional molecules [15]. These data suggest that the targeting of TIPE2 may be a novel and viable approach for NK cell-based immunotherapy.

In this study, we evaluated the expression of TNFAIP8L2 across different tumor types and correlated the level of expression with the prognosis. We further explored the relationship between TNFAIP8L2, cancer stem cell index and immune cell infiltration. Our data could provide novel insights into the expression of TNFAIP8L2 across different cancers and highlight a potential mechanism for TNFAIP8L2 within the tumor microenvironment that may be exploited as a novel cancer immunotherapy.

## 2 Methods

### 2.1 Data preparation and bulk expression in tissues

Clinical data from patients with different tumor types were obtained from the The-Cancer-Genome-Atlas (TCGA) including patient age, gender, grade and stage of disease. Gene expression data were obtained from the Genotype-Tissue Expression (GTEx) and TCGA databases (https://xenabrowser.net/datapages/). These data were merged using the Perl package in R to generate a matrix file to compare TNFAIP8L2 expression in normal and tumor samples. Statistical analysis was performed using a Student’s *t*-test. The ConsensusPathDB, Human Protein Atlas (HPA) and GTEx datasets were used to analyze TNFAIP8L2 RNA expression in different tissues and the level of protein expression was analyzed using the FANTOM5 dataset [16].

### 2.2 Single-cell expression analysis

The Single-Cell-Type section of the HPA included single-cell RNA-sequencing (scRNAseq) data from 25 human tissues and analysis of all protein-coding genes of 444 different cell clusters in 15 individual cell groups (https://www.proteinatlas.org/single + cell + type) [17]. The expression of genes in each of the cell types was visualized by UMAP plots and bar charts. The following criteria were used for gene selection:

1) The scRNAseq was performed in suspended cells that had not been pre-enriched for other cell types.
2) The sequencing data sequenced was > 20 million read counts.
3) The number of cells sequenced was ≥ 4,000 cells.
4) The correlation between pseudo- and real-expression profiles was significant.

The level of confidence was taken as the fraction of times a gene was assigned to the cluster in a repeated clustering and therefore reflected how strongly it was associated with the cluster. A confidence level of 1 indicated that the gene was assigned to a particular cluster in all of the repeated clusters.

### 2.3 Immune cell type specificity analysis

Immune cell type specificity analysis was performed using the immune-cell section of the protein atlas (https://www.proteinatlas.org/immune + cell). The peripheral blood immune cell lineages in this analysis included T-cells, granulocytes, NK-cells, dendritic cells, B-cells, monocytes, progenitors and total peripheral-blood-mononuclear-cell (PBMC). The level of TNFAIP8L2 expression in different blood cell lineages was analyzed using data from the HPA and Monaco datasets.

### 2.4 Subcellular localization of TIPE2

Immunofluorescence microscopy analysis of TIPE2 identified the subcellular section of the protein atlas (https://www.proteinatlas.org/ subcellular). The localization of TIPE2 in U2OS cells was displayed in green, the nucleus was shown in red and microtubules were shown in red. The atlas TIPE2 antibody was obtained from Sigma-Aldrich (HPA062742, Rabbit) and was used at a concentration of 0.151 mg/ml.

### 2.5 Prognostic analysis of TIPE2 expression

Univariate Cox regression analyses of TIPE2 expression in all cancers types were performed using the forest-plot packages in R to evaluate the prognostic indicators including overall survival (OS), disease-free-interval (DFI), progression-free-interval (PFI) and disease-specific-survival (DSS). Kaplan-Meier analysis was used to examine the significance of TIPE2 expression in predicting these prognostic indicators across different cancer types.

### 2.6 Stemness features of TIPE2 expression

The TNFAIP8L2 expression data were extracted from TCGA dataset and the tumor stemness score was calculated from the methylation signatures for each tumor that were obtained from previous studies [18]. The stemness indices of the samples and gene expression data were integrated and a log2(x + 0.001) transformation was performed on each of the expression values. Pearson’s analysis was performed to determine correlations between the tumor stemness score and TNFAIP8L2 expression using R software.

### 2.7 Immune cell infiltration

Immune cell infiltration was analyzed using the Estimate, xCELL and Tumor Immune Estimation Resource (TIMER) databases [19]. The Estimate dataset calculated the pseudo-immune score of each sample as a novel biomarker of immune cell infiltration in cancers. The xCELL and TIMER datasets provided the immune cell index transformed by the RNA-seq of samples. The correlation coefficients of the immune infiltration scores or immune infiltration cells with TNFAIP8L2 RNA expression were analyzed by Pearson’s correlation. All visualized results were generated using the ggplot2 package in R.

### 2.8 Gene-Set-Enrichment analysis

Samples were divided into high and low TNFAIP8L2 expression groups. The differentially expressed genes were selected for gene-set-enrichment-analysis (GSEA) using the clusterProfiler package in R. The significant pathways (P < 0.05) involved in the KEGG and HALLMARK pathways are displayed in Table S1.

### 2.9 The encyclopedia of RNA interactomes (ENCORI)

The ENCORI database was used to construct a comprehensive pan-cancer expression map and interaction network that contained > 10 cancer types. The overall survival was used to examine the efficacy of OS and analyzed using the starbase dataset (https://starbase.sysu.edu.cn/panCancer.php).

### 2.10 Statistical analyses

A Student’s *t*-test was used to compare TNFAIP8L2 expression between normal and tumor tissues and to analyze the significance of these characteristics in cancers. All correlation coefficients and visualized results were generated using the ggplot2 package in R. P-values < 0.05 were considered statistically significant (*= P < 0.05; ** = P < 0.01; *** = P < 0.001).

## 3 Results

### 3.1 Bulk and Single-cell expression of TNFAIP8L2

**Fig. 1:**
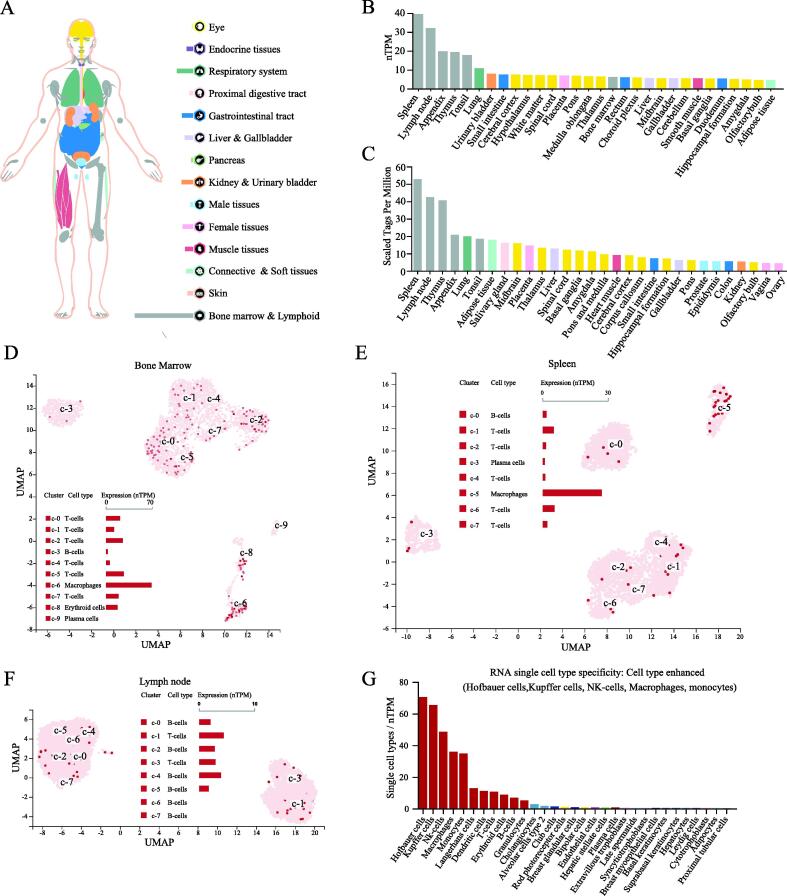
TNFAIP8L2 expression in normal tissues. (A) TNFAIP8L2 RNA expression in healthy tissues or organs from the Human Protein Atlas project. Different colors correspond to different tissues and organs. (B) RNA expression of TNFAIP8L2 in healthy tissues or organs from the ConsensusPathDB database. (C) TIPE2 protein expression in healthy tissues or organs from the FANTOM5 dataset. (D-F) Single-cell analysis of TNFAIP8L2 expression in the bone marrow (D), spleen (E) and lymph nodes (F). The red point re[resented different cells in clusters. Clusters were showed in coded rank by number 0 – 9. The expressions of cells in different clusters were showed by histogram. (G) TNFAIP8L2 expression in all cells at the single cell level. (For interpretation of the references to colour in this figure legend, the reader is referred to the web version of this article.)

TIPE2 is an important regulatory molecule in the immune system. We first evaluated the expression of TNFAIP8L2 in normal human tissues. We found that TNFAIP8L2 was most highly expressed in the bone marrow and lymphoid tissues, followed by the lungs, the gastrointestinal tract, kidney & urinary bladder (Fig. 1A). The RNA expression of TNFAIP8L2 was verified in the Consensus PathDB, HPA and GTEx datasets. These data showed that TNFAIP8L2 expression was highest in hematopoietic-related tissues/organs, especially the spleen and lymphoid (Fig. 1B and S1). Analysis of TIPE2 protein expression in human tissues/organs from the FANTOM5 dataset was consistent with results from the RNA expression analysis. The 5 human tissues/organs with the highest expression of TNFAIP8L2 were the spleen, lymph nodes, thymus, appendix and lungs (Fig. 1C).

To further refine the expression of TNFAIP8L2 in hematopoietic cells, single-cell sequencing data from hematopoietic tissues were analyzed. These data showed that macrophages expressed higher levels of TNFAIP8L2 compared to other immune cells in the bone marrow and spleen (Fig. 1D and 1E). In the lymph nodes, TNFAIP8L2 was more highly expressed in T-cells compared to B-cells (Fig. 1F). Next, we took the RNA single cell type specificity of all tissues together and found the TNFAIP8L2 expression was enhanced in Hofbauer cells, Kupffer cells, NK-cells, macrophages and monocytes (Fig. 1G and S2).

**Fig. 2:**
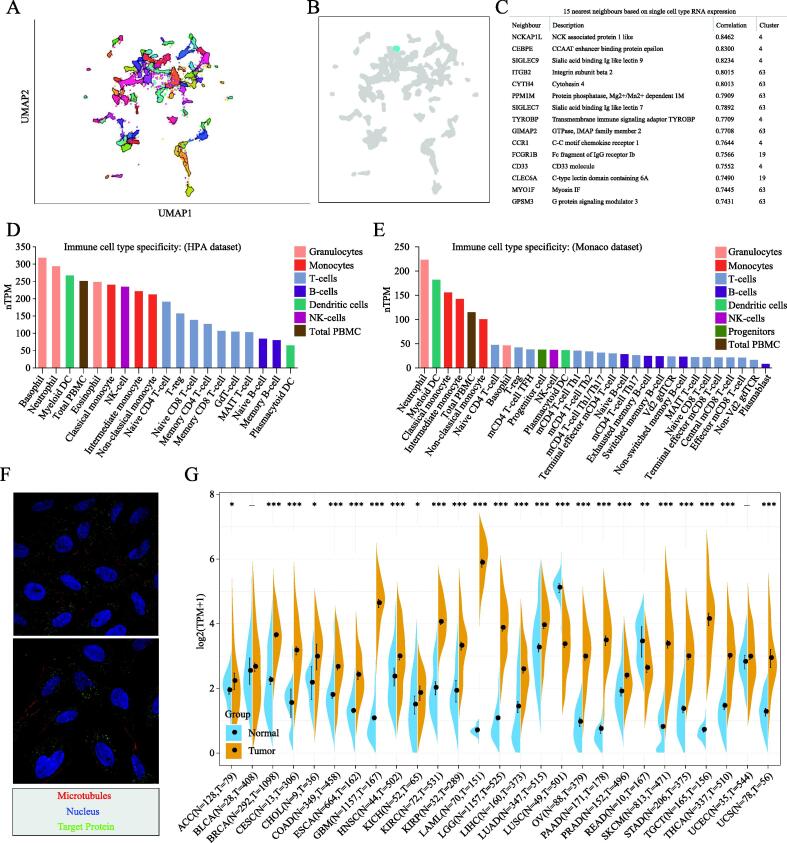
TNFAIP8L2 expression in tumors. (A) UMAP analysis of TNFAIP8L2 expression using single-cell RNA sequencing data from HPA. Different colors means different clusters. (B) The significant cluster 4 associated with TNFAIP8L2 expression among all the clusters. (C) The top 15 nearest neighbours based on single cell type RNA expression. (D) TNFAIP8L2 expression in immune cells from the HPA database. Different colors correspond to the different immune cells. (E) TNFAIP8L2 expression in immune cells from the Monaco database. (F) Subcellular localization of TIPE2 by immunofluorescence microscopy. Red indicates microtubules; blue indicates the nucleus; green indicates TIPE2. (G) TNFAIP8L2 expression in cancers from TCGA and the GETx database. (For interpretation of the references to colour in this figure legend, the reader is referred to the web version of this article.)

The 68 gene clusters resulting from the Louvain clustering of gene expression across all single cell types are displayed by UMAP and shown in Fig. 2A. The main specificity and reliability of cells with TNFAIP8L2 are listed in Table S2. Single-cell analysis indicated that TNFAIP8L2 was part of cluster 4 Macrophages of the innate immune response with a confidence level > 0.63 (Fig. 2B). Based on the single cell type RNA expression analysis shown in Fig. 2C, 228 genes were found in this cluster and the top 15 nearest neighbors (Table S3). The immune cell type specificities of hematopoietic tissues were validated by HPA and Monaco datasets. The HPA dataset demonstrated that macrophages, basophils, neutrophils and myeloid dendritic cells had high levels of TNFAIP8L2 expression (Fig. 2D). The results of the Monaco database analysis were similar to those from the HPA dataset with neutrophils, myeloid dendritic cells and monocytes being the top hematopoietic immune cells with the highest level of TNFAIP8L2 expression (Fig. 2E).

### 3.2 TNFAIP8L2 expression and cancer prognosis

To explore the significance of TIPE2 in cancers, we first analyzed the subcellular localization of TIPE2. Immunofluorescence microscopy data suggested that TIPE2 localized to vesicles (Fig. 2F). We then analyzed TNFAIP8L2 expression in tumors and corresponding adjacent tissues. The expression data in the GTEx database indicated that TNFAIP8L2 was highly expressed in most tumors, particularly in glioblastoma multiforme (GBM), kidney chromophobe (KICH), acute myeloid leukemia (AML), brain lower grade glioma (LGG), ovarian serous cystadenocarcinoma (OV), stomach adenocarcinoma (STAD) and testicular germ cell tumors (TGCT). Also, we found that TNFAIP8L2 was expressed at low levels only in lung squamous cell carcinoma (LUSC) and rectum adenocarcinoma (READ) (Fig. 2G).

**Fig. 3:**
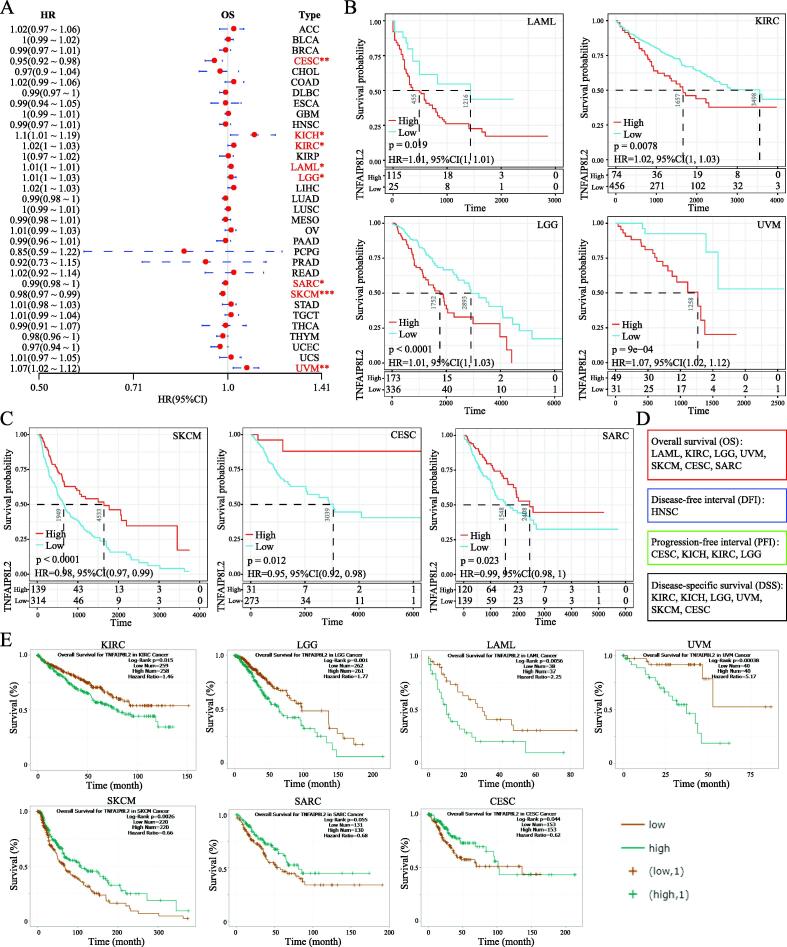
Prognosis analysis of TNFAIP8L2 in tumors. (A) Forest tree analysis of TNFAIP8L2 in all types of cancer. The HRs are shown to the left of the figure. (B) Survival analysis of TNFAIP8L2 on OS in LAML, LGG, KIRC and UVM. (C) Survival analysis of TNFAIP8L2 on OS in SKCM, CESC and SARC. (D) The summary of survival analysis of TNFAIP8L2 on OS, DFI, PFI and DSS in all cancers. *, p < 0.05; **, p < 0.01; ***, p < 0.001. (E) Kaplan–Meier analysis on OS in AML, LGG, KIRC, UVM, SKCM, CESC and SARC by ENCORI.

Next, we explored the prognostic value of TNFAIP8L2 in different tumor types. Forestplot analysis showed that TNFAIP8L2 expression predicted a poor prognosis of OS in LAML (HR = 1.01 (1–1.01)), LGG (HR = 1.01 (1–1.03)), KICH (HR = 1.1 (1.01–1.19)), KIRC (HR = 1.02 (1–1.03)) and UVM (HR = 1.07 (1.02–1.12)). Conversely, TNFAIP8L2 predicted a favorable prognosis in CESC (HR = 0.95 (0.92–0.98)), SARC (HR = 0.99 (0.98–1)) and SKCM (HR = 0.98 (0.97–0.99)) (Fig. 3A). The OS rates of patients with TNFAIP8L2 expressing tumors were validated by Kaplan–Meier analysis (Fig. 3B and 3C). We further analyzed the influence of TNFAIP8L2 expression on DFI, PFI and DSS. The results from univariate survival and Kaplan–Meier analysis is shown in Fig. 3d and S3-4. To validate our findings, we used the ENCORI OS analysis tool. Although the P-values were slightly different between the two methods, the conclusions were consistent with the previous results (Fig. 3E and Table S4).

### 3.3 The clinical phenotype of TNFAIP8L2 expressing tumors

**Fig. 4:**
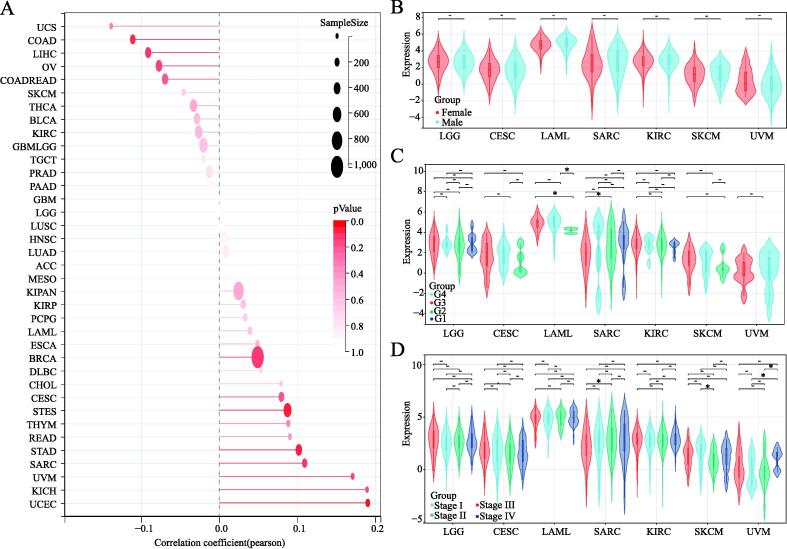
TNFAIP8L2 and clinical features in tumors. (A) The correlation of TNFAIP8L2 expression and age in cancers. (B) The expression value of TNFAIP8L2 between different genders in 7 tumors. Red indicates females and blue indicates males. (C) The relationship between disease grade and TNFAIP8L2 expression. Red = G3; blue = G4; Green = G2 and grey = G1. (D) The difference in TNFAIP8L2 expression at different stages of disease. Red = Stage III; blue = Stage IV; green = Stage II and grey = Stage I. (For interpretation of the references to colour in this figure legend, the reader is referred to the web version of this article.)

To gain insight into how TNFAIP8L2 expression affects prognosis, we first explored the relationship between TNFAIP8L2 expression and the basic clinical phenotypes in patients across all types of cancers. The correlation coefficient results indicated that there was no significant relationship between TNFAIP8L2 expression and age in major types of cancer (Fig. 4A). Also, no significant differences were found in TNFAIP8L2 expression in 7 types of cancer between males and females (Fig. 4B). We found similar levels of TNFAIP8L2 expression in different grades and stages of 7 types of tumors (Fig. 4C and 4D).

### 3.4 Stemness features of TNFAIP8L2 expressing tumors

**Fig. 5:**
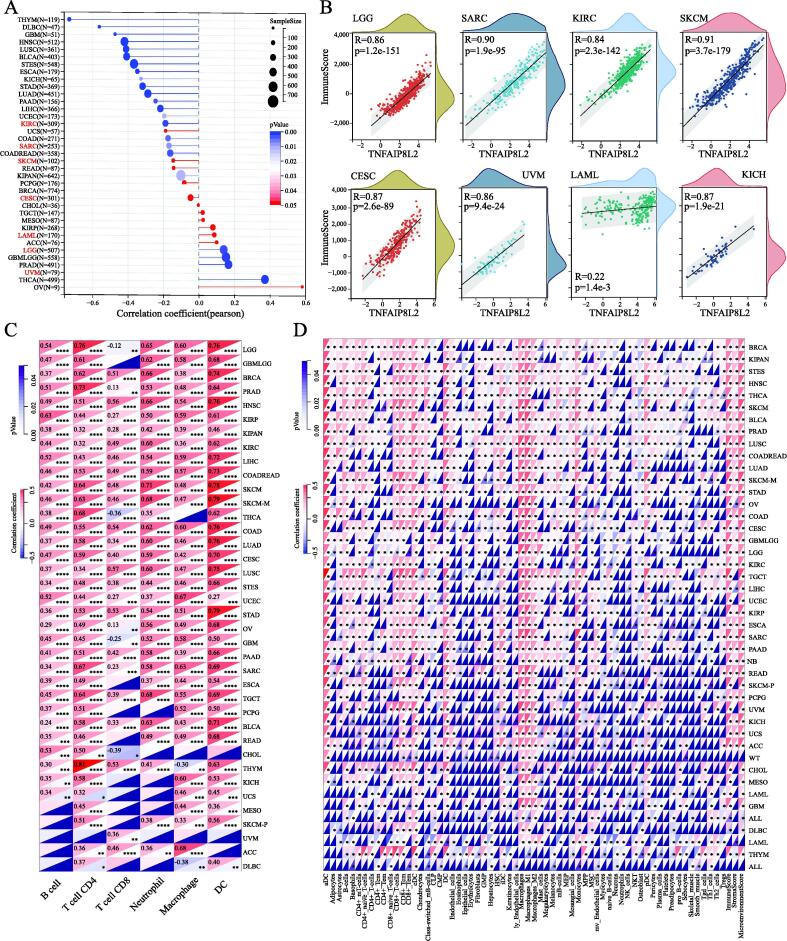
TNFAIP8L2, CSCs index and immune infiltration in cancers. (A) The correlation of TNFAIP8L2 expression and CSCs index in cancers. The target cancer types are marked in red. (B) Immune infiltration of TNFAIP8L2 in six types of cancers. The correlation value was calculated by Pearson analysis. (C-D) Validation of immune infiltration with TNFAIP8L2 in all cancers using Timer2 (C) and xCELL (D). (For interpretation of the references to colour in this figure legend, the reader is referred to the web version of this article.)

Stem cell index is considered a novel indicator of cancer development [20] and so we explored the correlation between cancer stem cell index and TNFAIP8L2 expression. Pearson analysis showed that TNFAIP8L2 expression was positively correlated with LAML and LGG but was negatively correlated with KIRC, SARC, SKCM and CESC (Fig. 5A). Combining the results of genetic prognosis, we found that the stem cell index and prognosis had consistent trends in LAML, LGG, SARC, SKCM and CESC, whilst opposing trends were observed in KIRC and UVM.

### 3.5 Immune cell infiltration of TNFAIP8L2 expressing cancers

We further explored the correlation between TNFAIP8L2 expression and immune cell infiltration. We calculated immune scores for each patient in each tumor based on gene expression using the R package ESTIMATE. Unexpectedly, we found that all 7 types of cancer displayed a significant positive correlation between TNFAIP8L2 expression and immune scores with correlation coefficients > 0.8 in all tumors except LAML (R = 0.22) (Fig. 5B).

Next, we further validated the immune cell infiltration in the other two datasets. The results of the TIMER2 dataset revealed that TNFAIP8L2 expression was positively correlated with the infiltration levels of B-cells, CD4^+^ T-cells, neutrophils, macrophages and dendritic cells, and partly negatively correlated with CD8^+^ T-cells (Fig. 5C). We used the deconvo-xCell method of the R package IOBR, to reassess the infiltration and immune scores in each tumor based on TNFAIP8L2 expression. Consistent with the TIMER analysis results, B-cells, CD4^+^ T-cells, neutrophils, macrophages and dendritic cells were positively related to TNFAIP8L2 expression. In addition, the correlation analyses with the xCell dataset indicated that other immune cells were closely related to the expression of TNFAIP8L2 (Fig. 5D). In total, the immune cell infiltrations induced by TNFAIP8L2 from all three different datasets were consistent. These results suggest that TNFAIP8L2 may contribute to increased macrophage and dendritic cell infiltration and could be of prognostic significance in most cancer types.

### 3.6 GSEA of TNFAIP8L2

Functionally, we evaluated the pathways associated with TNFAIP8L2 expression by GSEA. Samples were divided into two groups according to the level of TNFAIP8L2 expression. The results indicated that the metabolic synthesis pathways were significantly enriched in the TNFAIP8L2 low expression group (Table S1). The major metabolic synthesis pathways included Aminoacyltrna biosynthesis, Glycosyl phosphatidylinositolgpianchor biosynthesis, Propanoate metabolism, Valine leucine and isoleucine_biosynthesis, Pyruvate metabolism, Arginine and proline metabolism, N-glycan_biosynthesis and Terpenoid backbone biosynthesis (Figure S5-6). Also, several hematopoietic and immune-related pathways were significantly enriched in the high TNFAIP8L2 expression group. These data further demonstrate the importance and strong relevance of immune cell infiltration and TNFAIP8L2 expression.

## 4 Discussion

Immune checkpoint blockade therapy using targeted antibodies has shown promising clinical results in a range of tumor types [21], [22]. During prolonged cancer treatment, exhausted T-cells gradually lose their function during the process of *T*-cell exhaustion [2], [23], however, checkpoint blockade can promote the proliferation of tumor-infiltrating CD8^+^ T lymphocytes [4]. Currently, there are three common target sites for immune checkpoint therapy: PD-1, CTLA-4 and PD-L1 [8], [24], [25]. TIPE2 is encoded by TNFAIP8L2 and has been identified as a potential checkpoint molecule for NK cells maturation and anti-tumor immunity [26] and so targeting TIPE2 may be a potentially novel NK cell-based immunotherapy approach [3].

In this study, we analyzed the expression of TNFAIP8L2 in different tissues, especially hematopoietic tissues, by single-cell sequencing analysis. Our data indicated that TNFAIP8L2 was mainly highly expressed in Hofbauer cells, Kupffer cells, NK cells, macrophages and monocytes. These findings further validate the important immune function of TIPE2 in the regulation of the immune system. We evaluated the expression and prognostic significance of TNFAIP8L2 in different cancer types and found that as a negative immune regulator, TNFAIP8L2 was highly expressed in most tumors. Kaplan-Meier analysis of OS showed that TNFAIP8L2 expression was a risk factor in LAML, LGG, KIRC and UVM, yet was a protective factor in CESC, SARC and SKCM. For DSS, univariate Cox regression analysis indicated that TNFAIP8L2 expression was a risk factor for KICH, KIRC, LGG and UVM patients, yet was a protective factor in CESE and SKCM patients. DFI and PFI were used to reflect the importance of TNFAIP8L2 expression in patients. The DFI results demonstrated that TNFAIP8L2 expression is a risk for patients with HNSC and the PFI data showed that TNFAIP8L2 was a risk for patients with KICH, KIRCA and LGG but had a protective function for patients with CESC. Collectively, these results suggest that low TNFAIP8L2 expression is associated with anti-tumor function in most cancers.

Finally, we found that the TNFAIP8L2 expression had prognostic significance in only 7 tumor types in which it acted as a poor prognostic factor in LAML, LGG, KICH, KIRC and UVM. In CESC, SARC and SKCM, TNFAIP8L2 expression was a strong prognostic factor. We then explored the potential reasons for the differential prognosis based on TNFAIP8L2 expression in different tumors and we found no clear correlation between TNFAIP8L2 expression and the general clinical characteristics of tumor patients. These findings suggest that the observed prognostic differences may be at the cellular and molecular level.

Cancer stem cells (CSC) are characterized by self-renewal and resistance to therapy and play an important role in many cancer types [20]. Here, we calculated tumor cell to stem cell similarity by RNA expression and quantified it as the CSC index. Our results indicated that TNFAIP8L2 expression was significantly correlated with LAML, LGG, KIRC, SARC, SKCM and CESC. The higher expression of TNFAIP8L2 indicated a poorer prognosis in LAML and LGG which was positively correlated with the CSC index. A higher expression of TNFAIP8L2 indicated a favorable prognosis in SARC, SKCM and CESC which had a negative correlation with the CSC index. These data suggest that TNFAIP8L2 expression can impact disease prognosis by affecting the CSC index in LAML, LGG, SARC, SKCM and CESC. However, opposing data were observed in KIRC and UVM indicating that other mechanisms are involved.

TNFAIP8L2 is a potentially new immune checkpoint factor that functions to maintain immune homeostasis [27]. Our immune cell infiltration result indicated that TNFAIP8L2 might increase macrophage and dendritic cell infiltration in the tumor microenvironment and affect the prognosis in some cancers. In addition, functional GSEA analysis showed that metabolic synthesis pathways and hematopoietic and immune-related pathways were affected by TNFAIP8L2 expression. The immune cell infiltration data, CSC index and GSEA are complementary in demonstrating the prognostic value of TNFAIP8L2 expression in different cancer types.

In this study, we found that TNFAIP8L2 expression was associated with a poor prognosis in LAML, LGG, KICH, KIRC and UVM. These data suggest that TIPE2 inhibitors may have a synergistic therapeutic effect in these types of tumors. Due to the extensive distribution of targeted molecules, chemotherapy and targeted drug have different degrees of adverse reactions. CTLA-4 inhibitors are commonly associated with enteritis, hypophysis, and skin rashes whereas PD-1 inhibitors can cause immune pneumonia, muscle and joint pain, and hypothyroidism. According to the distribution of TIPE2 in systemic tissues, we speculated that TIPE2 inhibitors may have some side effects such as immune-related pneumonia, endocrine disorders, various types of digestive tract discomfort, and symptoms associated with a cytokine storm. We are planning to perform experiments to verify the influence of the CSC index on different tumor types and prognosis through a prospective control cohort clinical trial to verify the hypothesis presented in this study.

## Declaration of Competing Interest

The authors declare that they have no known competing financial interests or personal relationships that could have appeared to influence the work reported in this paper.
