# Supplementary figures and images for: Comprehensive analysis of tumor necrosis factor-α-inducible protein 8-like 2 (TIPE2): A potential novel pan-cancer immune checkpoint
Source: Comput Struct Biotechnol J. 2022 Sep 17;20:5226–34. doi: 10.1016/j.csbj.2022.09.021 (PMC9508481; doi:10.1016/j.csbj.2022.09.021)

Figure S1

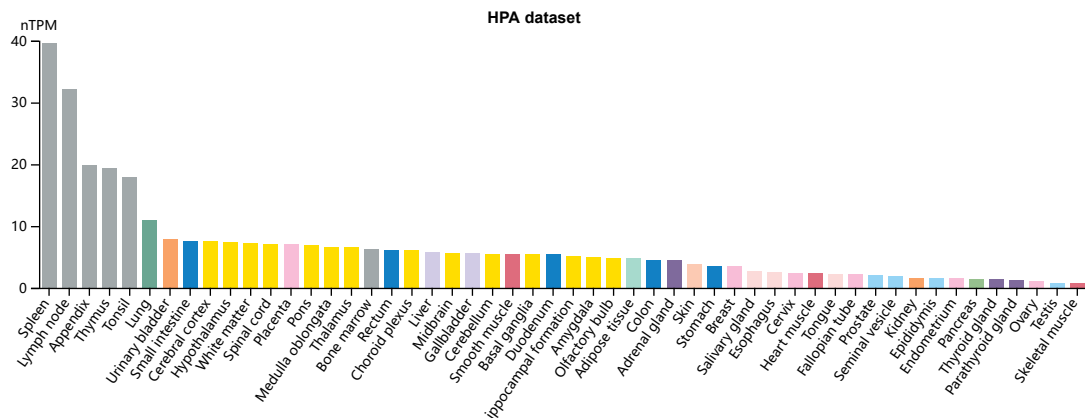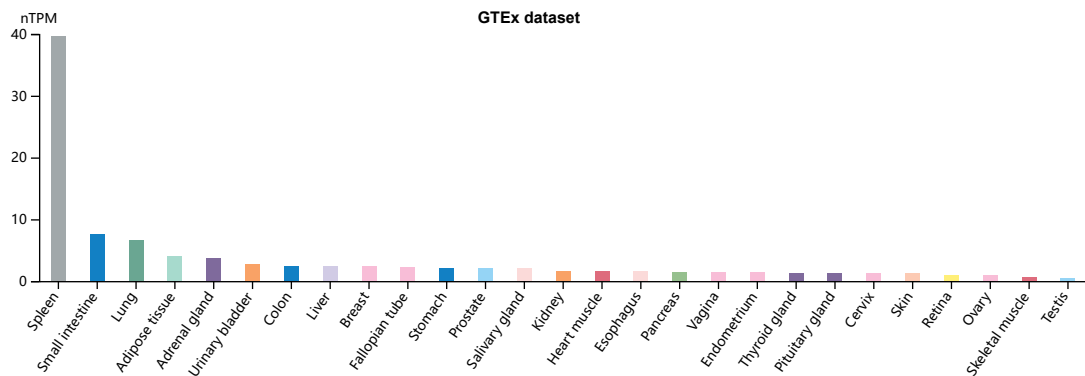

Supplement: Supplementary data 1 [file mmc1.pdf]

Figure S2

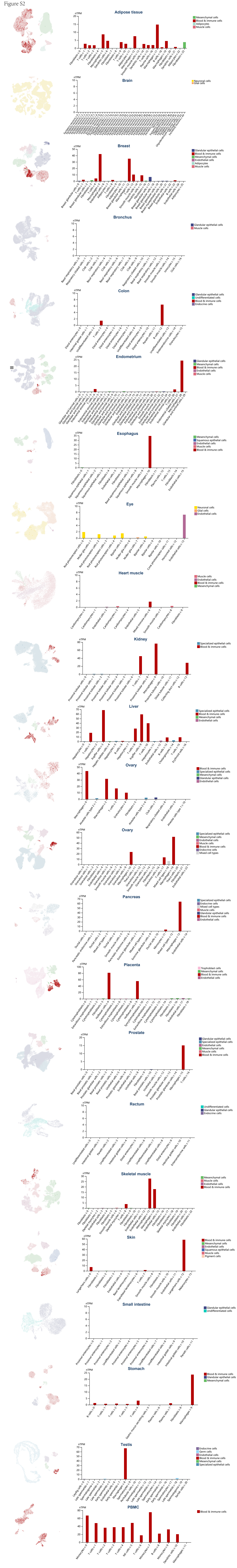

Supplement: Supplementary data 2 [file mmc2.pdf]

Figure S4  
DFI

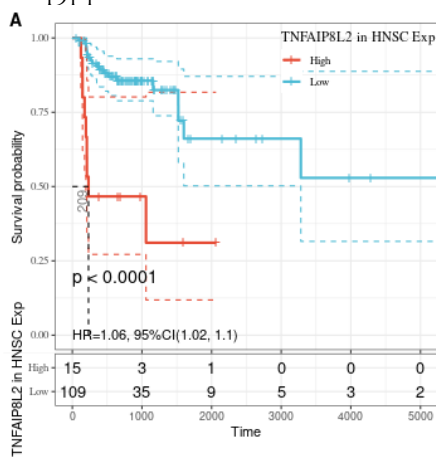

DSS

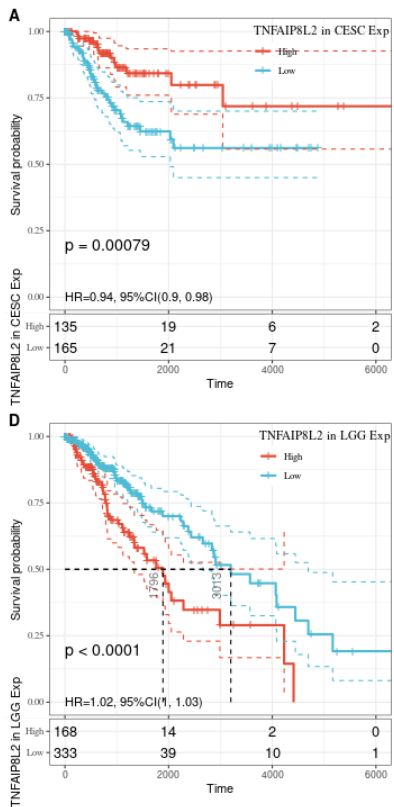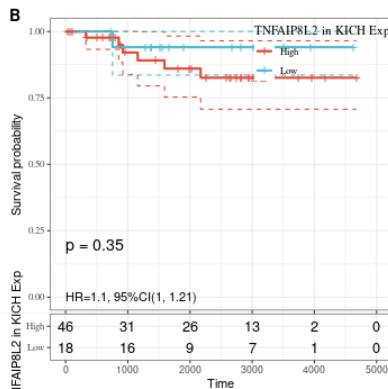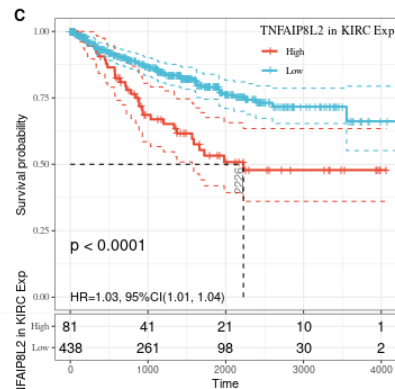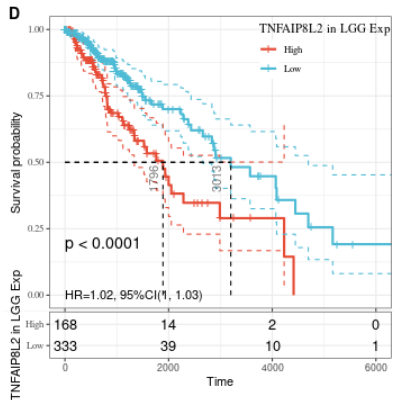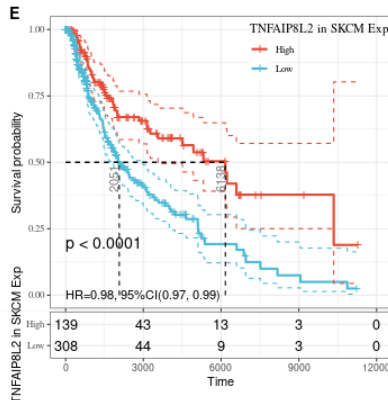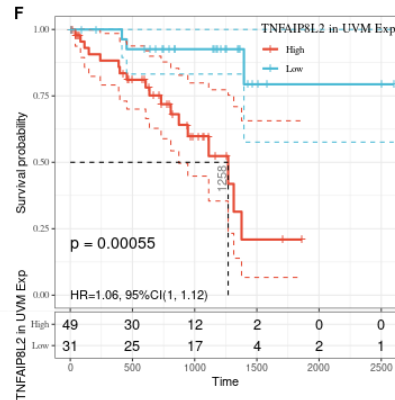

PFI

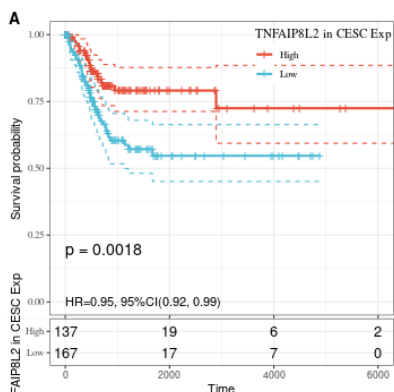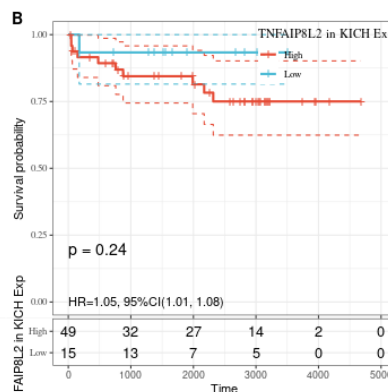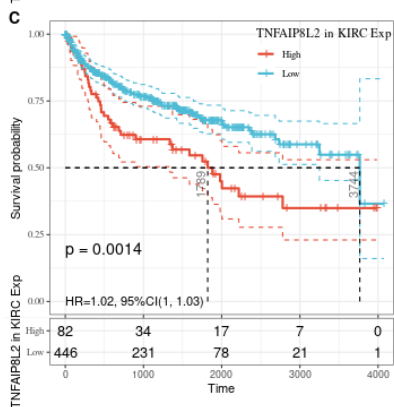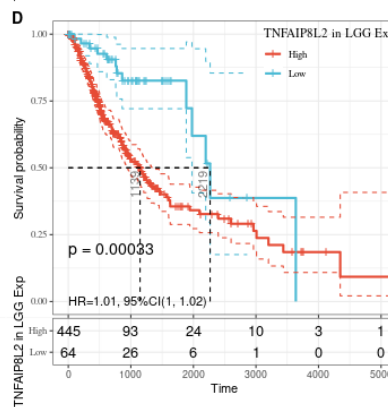

Supplement: Supplementary data 4 [file mmc4.pdf]

Table: Snapshot of enrichment results

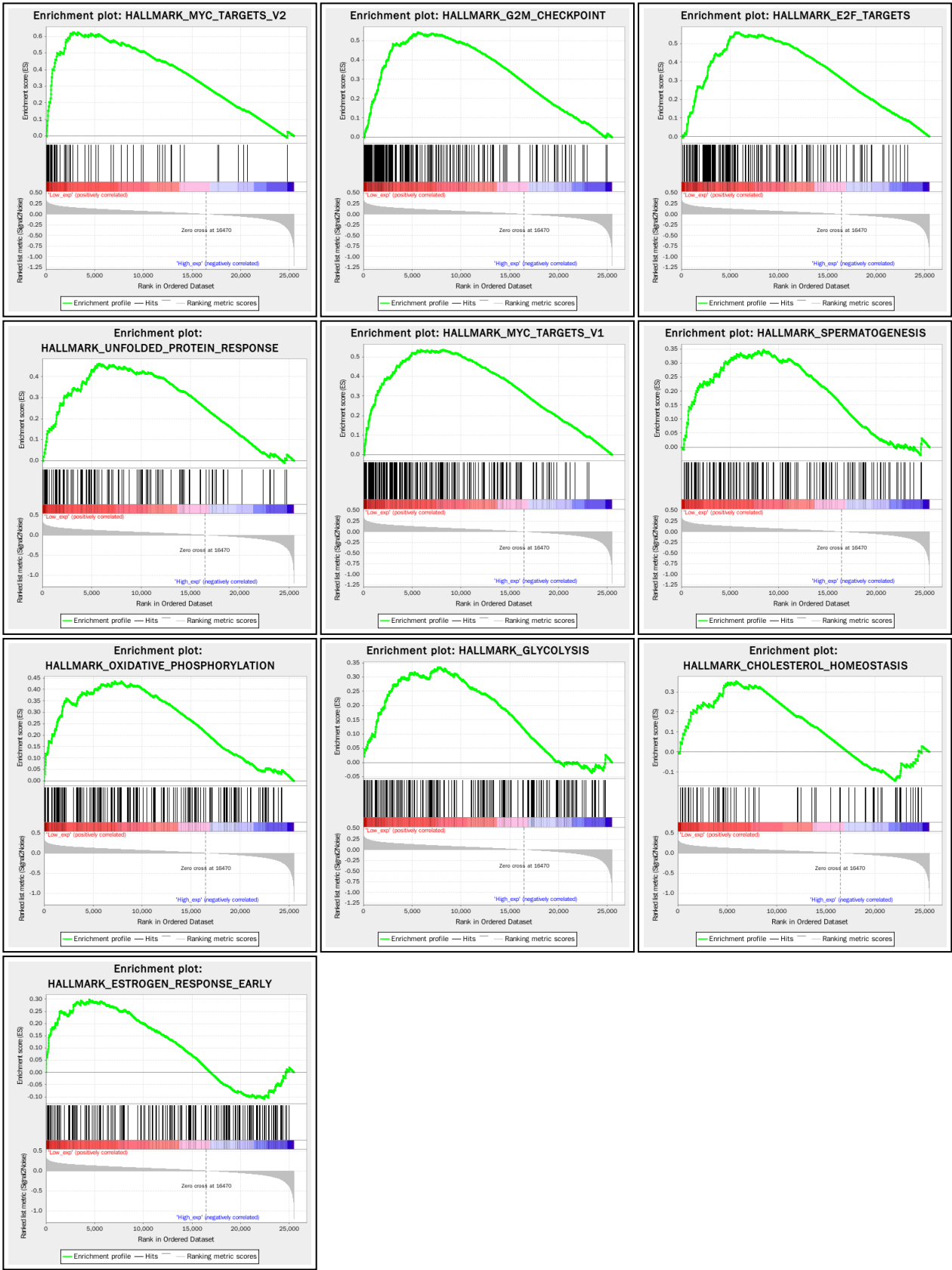

Supplement: Supplementary data 5 [file mmc5.pdf]
