# Supplementary material for: Comprehensive analysis of tumor necrosis factor-α-inducible protein 8-like 2 (TIPE2): A potential novel pan-cancer immune checkpoint
Source: Comput Struct Biotechnol J. 2022 Sep 17;20:5226–34. doi: 10.1016/j.csbj.2022.09.021 (PMC9508481; doi:10.1016/j.csbj.2022.09.021)

Figure S3

DFI

	HR	P Value
ACC	1.03(0.98 ~ 1.09)	0.2300
BLCA	0.96(0.91 ~ 1.01)	0.0900
BRCA	0.99(0.96 ~ 1.01)	0.3000
CESC	0.96(0.92 ~ 1.01)	0.1100
CHOL	0.98(0.93 ~ 1.04)	0.5300
COAD	0.95(0.85 ~ 1.05)	0.3000
DLBC	0.99(0.96 ~ 1.02)	0.5600
ESCA	1.03(0.97 ~ 1.09)	0.3300
GBM	NA(NA ~ NA)	
HNSC	1.06(1.02 ~ 1.1)	0.0014
KICH	0.87(0.51 ~ 1.51)	0.6300
KIRC	0.98(0.93 ~ 1.03)	0.4600
KIRP	1.02(0.99 ~ 1.04)	0.1400
LAML	NA(NA ~ NA)	
LGG	0.99(0.96 ~ 1.02)	0.4300
LIHC	1(0.97 ~ 1.02)	0.7500
LUAD	0.99(0.97 ~ 1.01)	0.2900
LUSC	1(0.97 ~ 1.03)	0.9800
MESO	1.02(0.92 ~ 1.13)	0.7200
OV	0.99(0.96 ~ 1.02)	0.6900
PAAD	0.99(0.94 ~ 1.03)	0.5200
PCPG	0.6(0.26 ~ 1.38)	0.2300
PRAD	1(0.92 ~ 1.09)	0.9800
READ	1.02(0.82 ~ 1.28)	0.8400
SARC	1(0.99 ~ 1.01)	0.4400
SKCM	NA(NA ~ NA)	
STAD	1.01(0.96 ~ 1.06)	0.6700
TGCT	0.98(0.96 ~ 1.01)	0.1400
THCA	1(0.95 ~ 1.05)	0.9300
THYM	NA(NA ~ NA)	
UCEC	0.96(0.92 ~ 1.01)	0.0950
UCS	1(0.92 ~ 1.1)	0.9500
UVM	NA(NA ~ NA)	

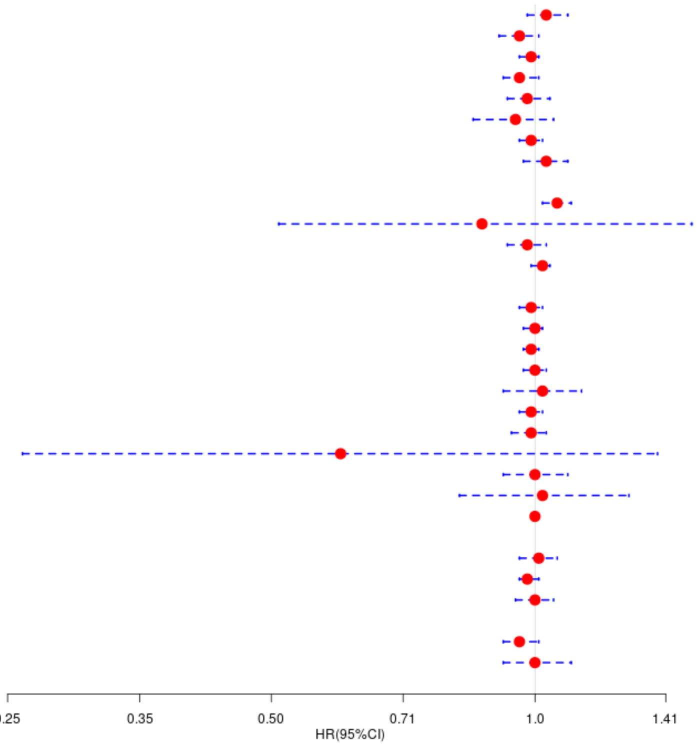

DSS

	HR	P Value
ACC	1(0.95 ~ 1.06)	0.93000
BLCA	1(0.99 ~ 1.02)	0.75000
BRCA	0.99(0.97 ~ 1.01)	0.37000
CESC	0.94(0.9 ~ 0.98)	0.00490
CHOL	0.96(0.87 ~ 1.05)	0.37000
COAD	1.03(0.99 ~ 1.07)	0.16000
DLBC	0.99(0.98 ~ 1.01)	0.51000
ESCA	1.02(0.96 ~ 1.08)	0.52000
GBM	1(0.99 ~ 1.01)	0.77000
HNSC	0.98(0.95 ~ 1)	0.11000
KICH	1.1(1 ~ 1.21)	0.04500
KIRC	1.03(1.01 ~ 1.04)	0.00110
KIRP	0.99(0.96 ~ 1.03)	0.77000
LAML	NA(NA ~ NA)	
LGG	1.02(1 ~ 1.03)	0.01100
LIHC	1.01(0.99 ~ 1.04)	0.30000
LUAD	0.99(0.97 ~ 1)	0.09000
LUSC	1(0.98 ~ 1.02)	0.96000
MESO	0.99(0.96 ~ 1.02)	0.52000
OV	1(0.98 ~ 1.02)	0.77000
PAAD	0.99(0.96 ~ 1.02)	0.43000
PCPG	0.96(0.69 ~ 1.32)	0.79000
PRAD	0.87(0.6 ~ 1.27)	0.47000
READ	0.94(0.8 ~ 1.11)	0.47000
SARC	0.99(0.98 ~ 1)	0.05400
SKCM	0.98(0.97 ~ 0.99)	0.00017
STAD	1.02(0.99 ~ 1.05)	0.28000
TGCT	1.02(0.99 ~ 1.04)	0.22000
THCA	0.89(0.75 ~ 1.07)	0.23000
THYM	0.98(0.95 ~ 1.01)	0.27000
UCEC	0.96(0.92 ~ 1.01)	0.08500
UCS	0.99(0.94 ~ 1.04)	0.64000
UVM	1.06(1 ~ 1.12)	0.03800

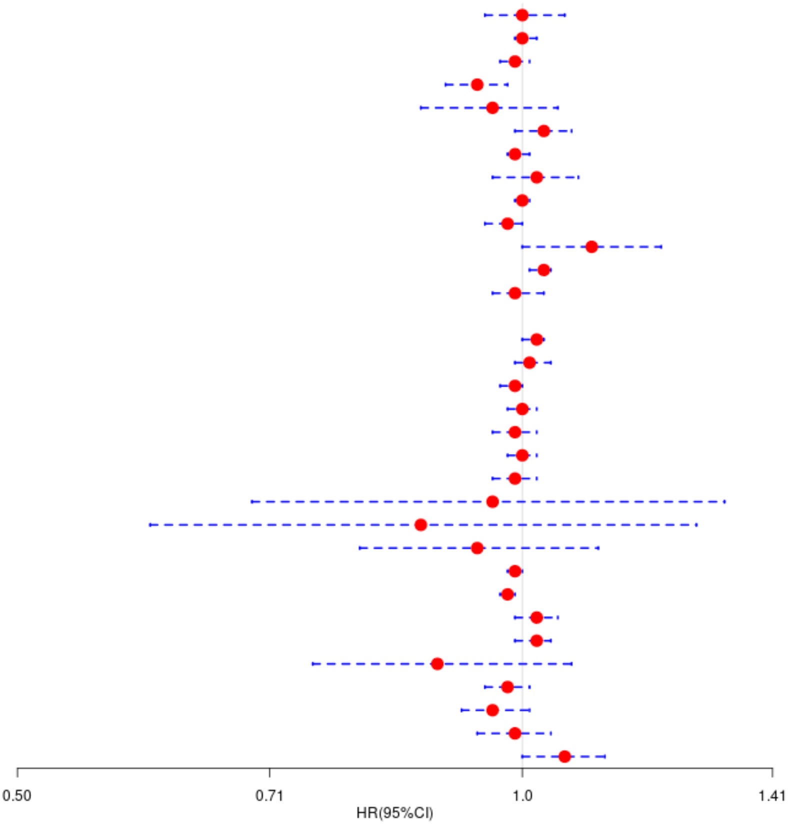

PFI

	HR	P Value
ACC	0.99(0.94 ~ 1.03)	0.5500
BLCA	0.99(0.98 ~ 1.01)	0.4300
BRCA	0.99(0.97 ~ 1)	0.1200
CESC	0.95(0.92 ~ 0.99)	0.0048
CHOL	0.92(0.84 ~ 1.02)	0.1200
COAD	1(0.97 ~ 1.04)	0.8400
DLBC	0.99(0.98 ~ 1.01)	0.3000
ESCA	1(0.96 ~ 1.05)	0.8500
GBM	1.01(1 ~ 1.02)	0.2000
HNSC	0.99(0.97 ~ 1.01)	0.2400
KICH	1.05(1.01 ~ 1.08)	0.0150
KIRC	1.02(1 ~ 1.03)	0.0140
KIRP	1(0.98 ~ 1.02)	0.8100
LAML	NA(NA ~ NA)	
LGG	1.01(1 ~ 1.02)	0.0160
LIHC	1.01(0.99 ~ 1.03)	0.5600
LUAD	0.99(0.98 ~ 1)	0.1600
LUSC	1(0.98 ~ 1.02)	0.8000
MESO	0.99(0.97 ~ 1.01)	0.4800
OV	1(0.98 ~ 1.02)	0.8400
PAAD	0.99(0.97 ~ 1.01)	0.3800
PCPG	0.97(0.84 ~ 1.12)	0.7100
PRAD	1.03(1 ~ 1.07)	0.0750
READ	0.96(0.87 ~ 1.05)	0.3400
SARC	1(0.99 ~ 1)	0.1800
SKCM	1(0.99 ~ 1)	0.2700
STAD	1.01(0.98 ~ 1.03)	0.6000
TGCT	0.99(0.97 ~ 1.01)	0.1900
THCA	0.99(0.95 ~ 1.03)	0.5200
THYM	1(0.98 ~ 1.01)	0.5900
UCEC	0.98(0.95 ~ 1)	0.0710
UCS	0.98(0.93 ~ 1.02)	0.3200
UVM	1.04(0.99 ~ 1.1)	0.1300

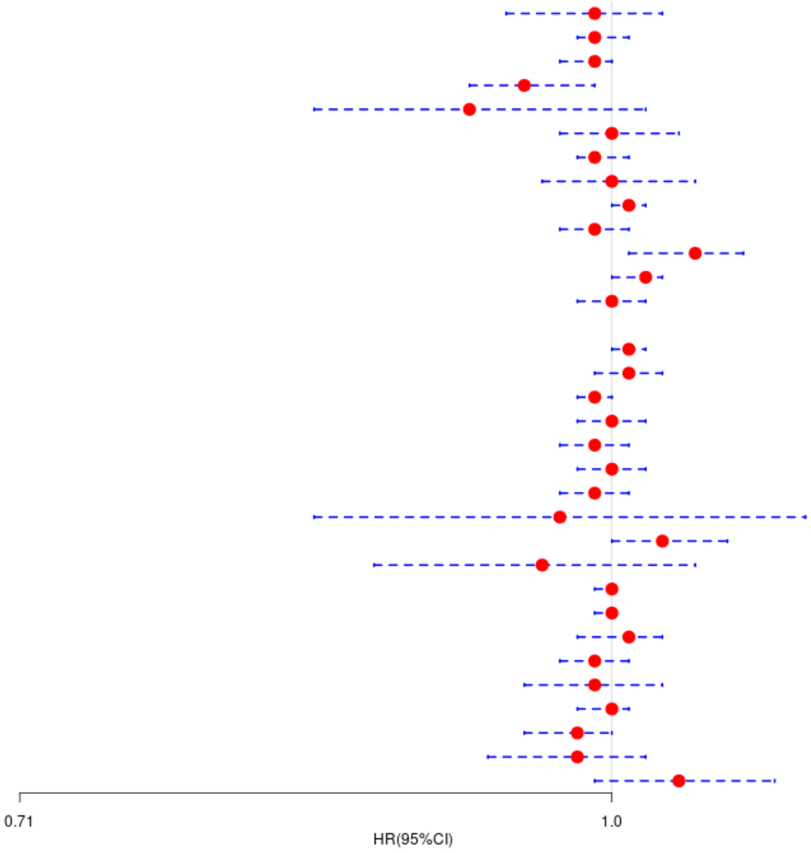

Supplement: Supplementary data 3 [file mmc3.pdf]
