# Supplementary material for: Comprehensive analysis of tumor necrosis factor-α-inducible protein 8-like 2 (TIPE2): A potential novel pan-cancer immune checkpoint
Source: Comput Struct Biotechnol J. 2022 Sep 17;20:5226–34. doi: 10.1016/j.csbj.2022.09.021 (PMC9508481; doi:10.1016/j.csbj.2022.09.021)

Figure S6

Enrichment in phenotype High_exp (257 samples)

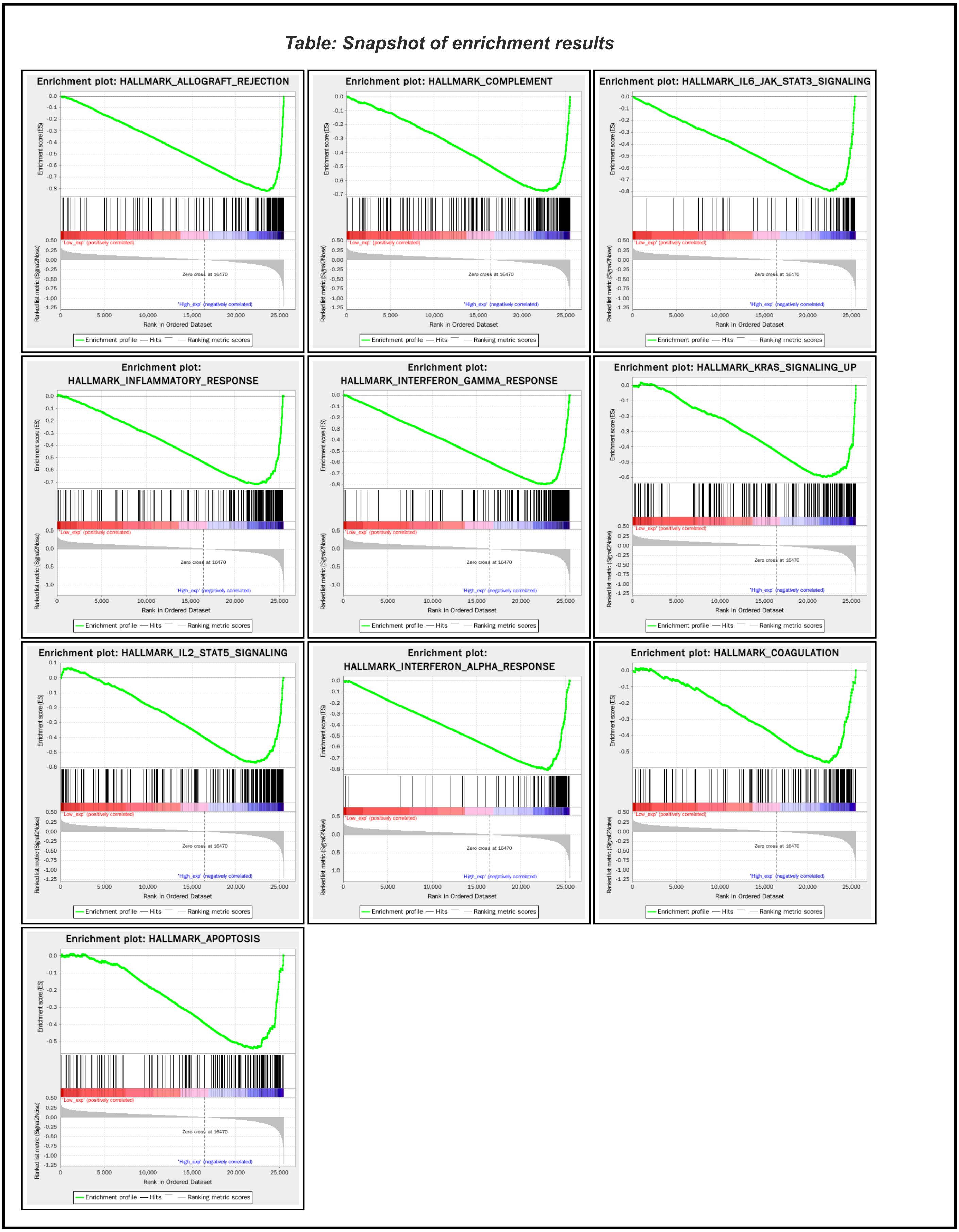

Table: Snapshot of enrichment results

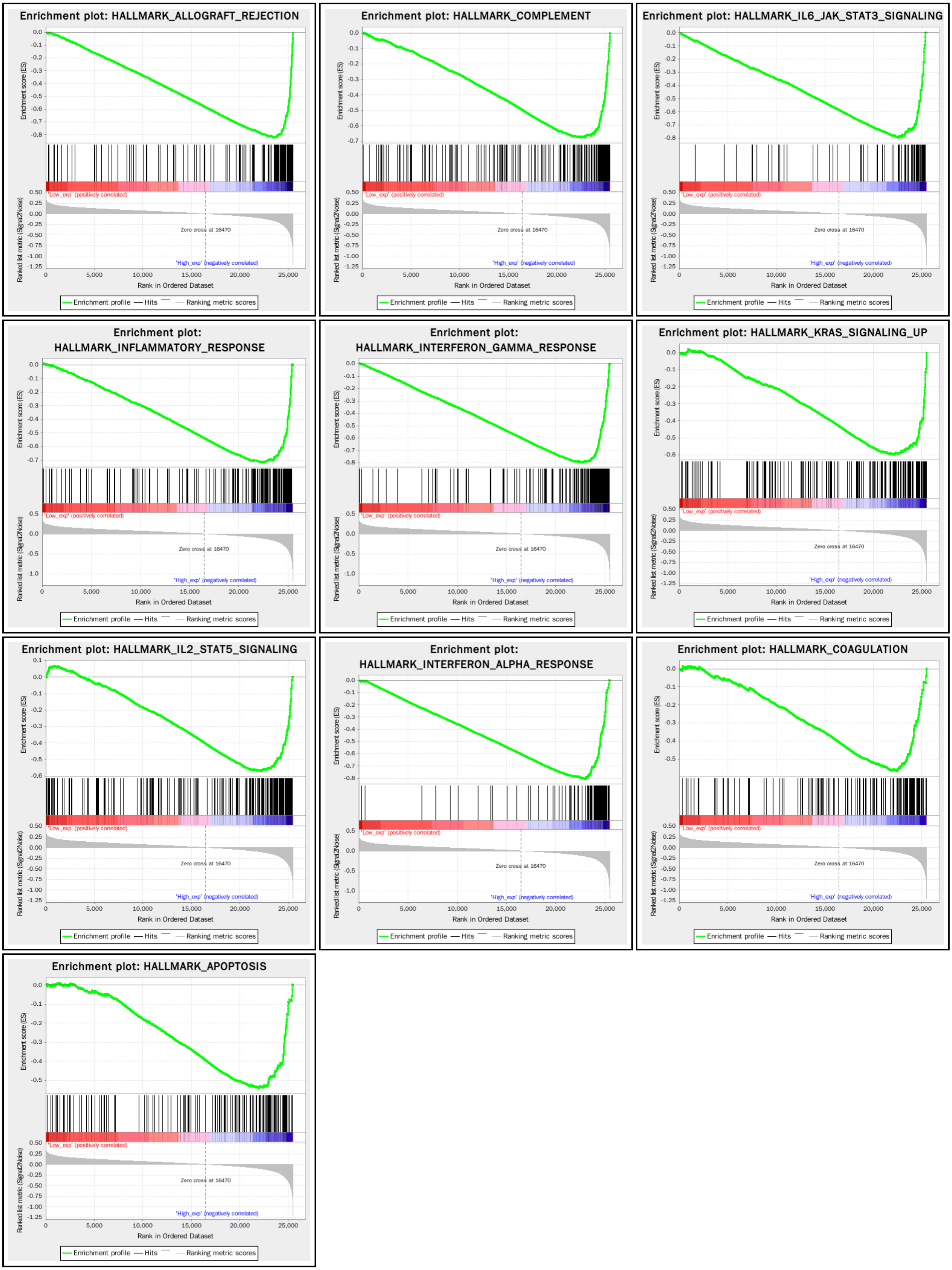

Table: Snapshot of enrichment results

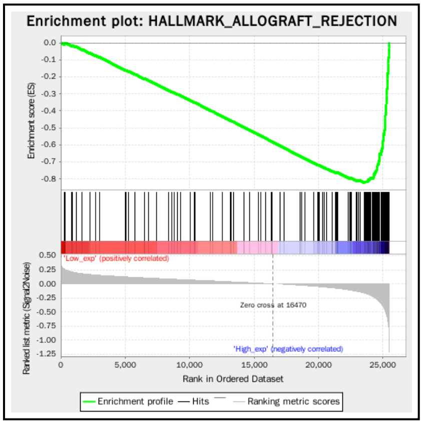

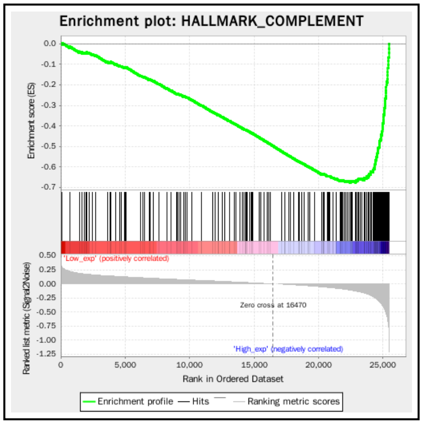

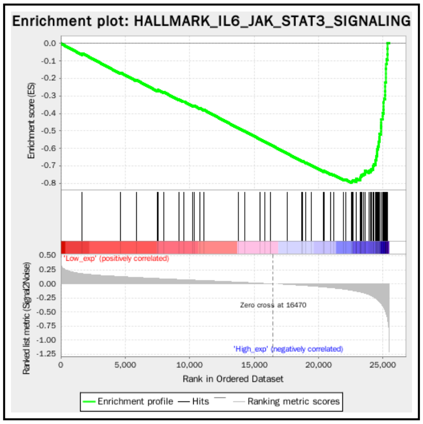

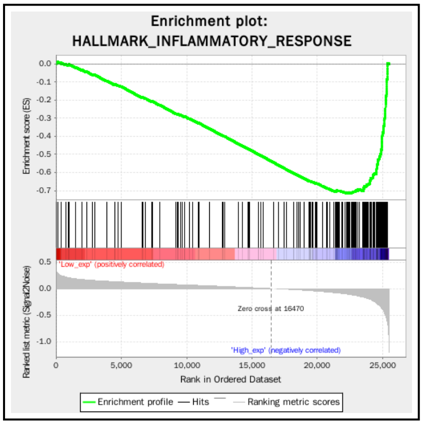

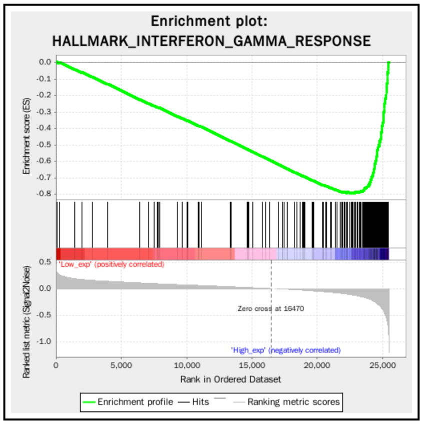

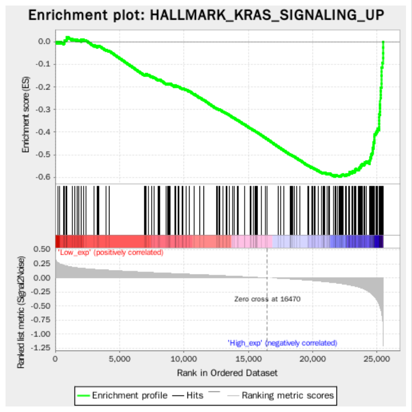

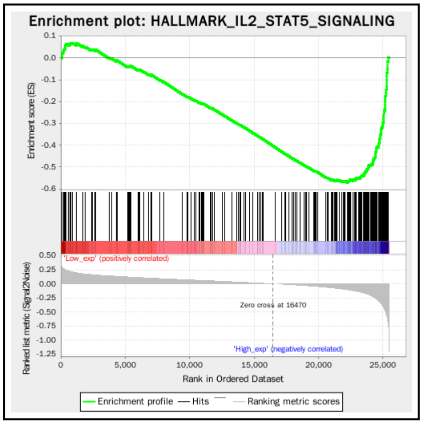

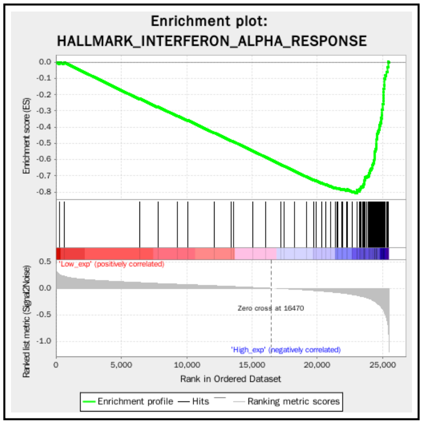

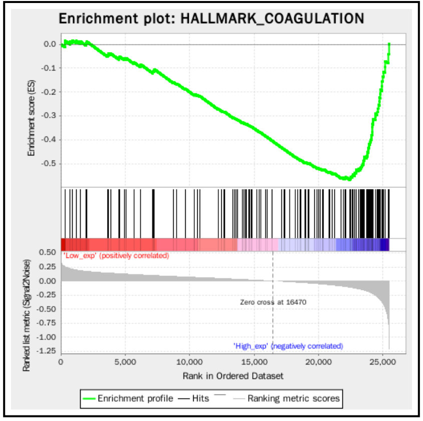

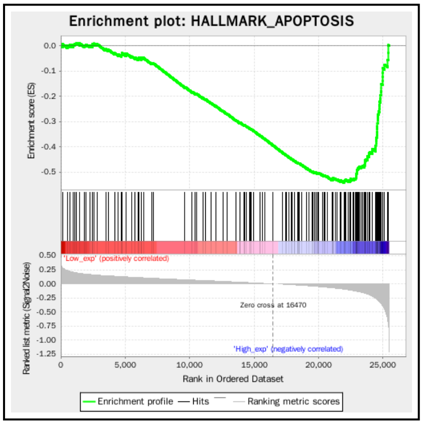

Table: Snapshot of enrichment results

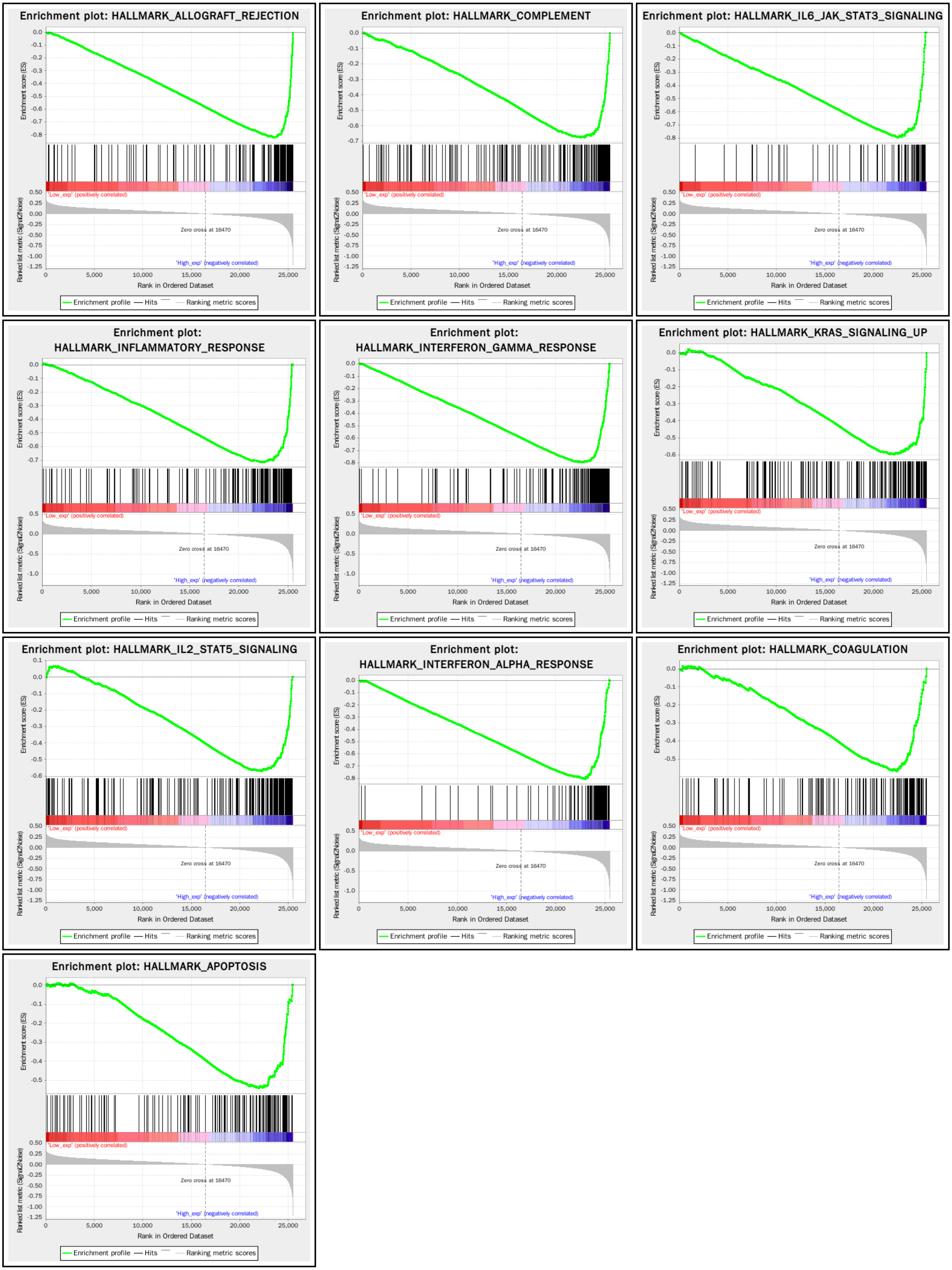

Table: Snapshot of enrichment results

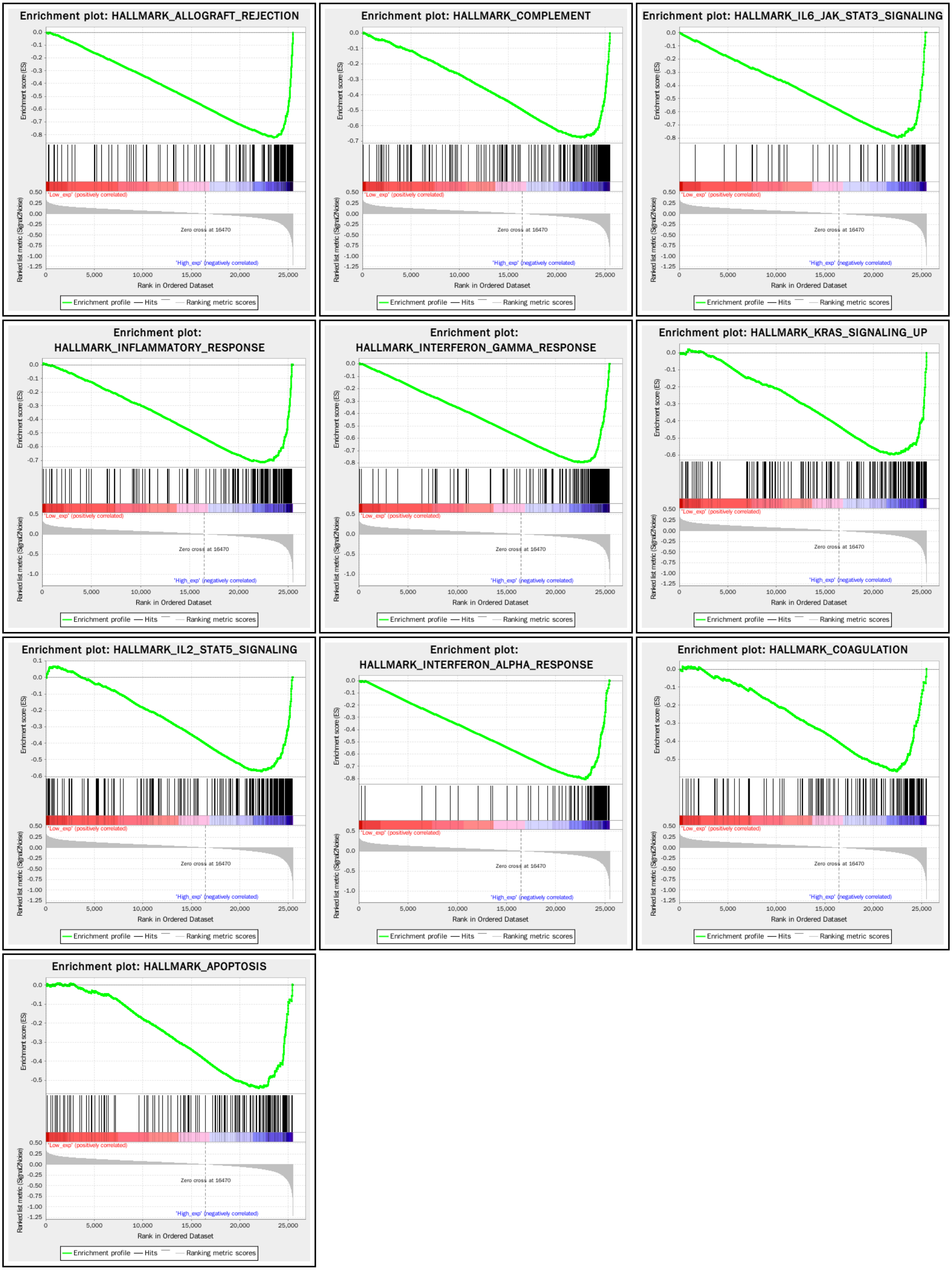

Table: Snapshot of enrichment results

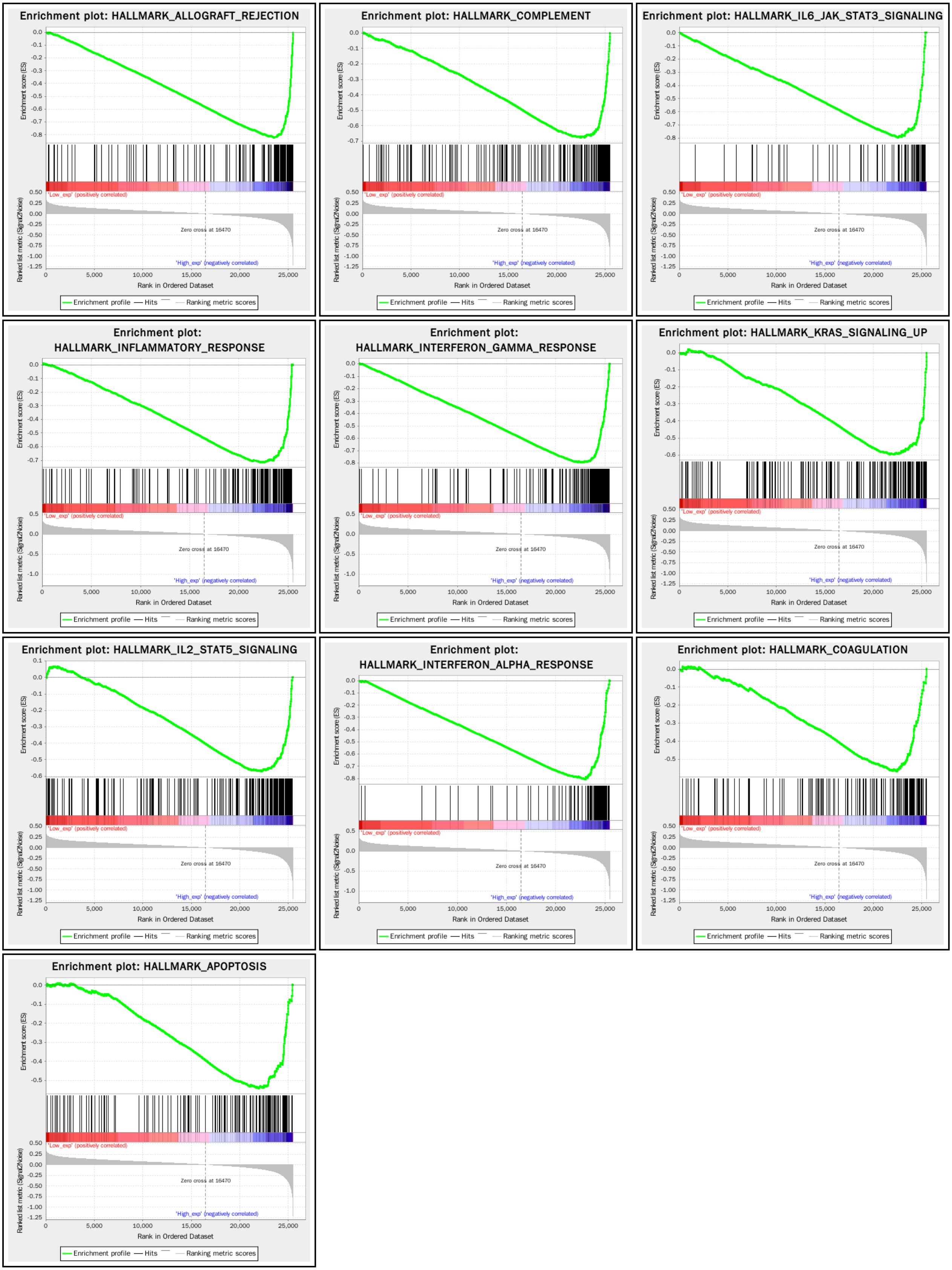

Supplement: Supplementary data 6 [file mmc6.pdf]
